# New cell lines derived from larvae of the neotropical fruit fly *Drosophila willistoni* persistently infected with *Wolbachia*

**DOI:** 10.1080/19336934.2026.2690767

**Published:** 2026-06-22

**Authors:** Lesley Bell-Sakyi, Elina Koivisto, Anton Strunov, Catherine Hartley, Jing Jing Khoo, Ewa Chrostek, Aurélie Hua-Van, Benjamin L. Makepeace, Wolfgang J. Miller

**Affiliations:** a Department of Infection Biology and Microbiomes, Institute of Infection, Veterinary and Ecological Sciences, University of Liverpool, Liverpool, UK; b Department of Cell and Developmental Biology, Center of Anatomy and Cell Biology, Medical University of Vienna, Vienna, Austria; c Department of Evolution, Ecology and Behaviour, Institute of Infection, Veterinary and Ecological Sciences, University of Liverpool, Liverpool, UK; d Department of Evolution, Genomes, Behavior, Ecology (EGCE), University Paris-Saclay, Gif-sur-Yvette, France

**Keywords:** Insect, cell line, *Drosophila willistoni*, chromosome, symbiont, Wolbachia

## Abstract

Although several hundred continuous cell lines have been generated from *Drosophila* spp. fruit flies over the past half-century, nearly all are derived from a single species, *Drosophila melanogaster*, and none are derived from neotropical flies. To address this deficit, a simplified protocol was used to generate three primary cell cultures from larvae of *Drosophila willistoni* originating from Costa Rica. All three primary cultures developed into continuous cell lines, and all three cell lines, designated DWL/LULS68, DWL/LULS70 and DWL/LULS72, were found to be persistently infected with the bacterial endosymbiont *Wolbachia*. Sublines free of *Wolbachia* were generated from all three cell lines by prolonged tetracycline treatment. Molecular analysis, karyotyping and fluorescence *in situ* hybridization confirmed species origin of the cells as *D. willistoni* and identified the *Wolbachia* as the strain *w*Wil. *Wolbachia w*Wil was successfully transferred from *D. willistoni* cells to heterologous cell lines derived from the sand fly *Lutzomyia longipalpis*, the biting midge *Culicoides sonorensis* and the tsetse fly *Glossina morsitans*, but not to cell lines derived from ticks or triatomine bugs. The new *D. willistoni* cell lines are expected to facilitate many aspects of research into this species and its bacterial symbionts.

## Introduction

The first cell lines derived from a fruit fly, *Drosophila melanogaster*, were established from insect embryos in laboratories in the Soviet Union, France and USA in the late 1960s–early 1970s [1]. The Cellosaurus website lists 386 cell lines, sublines and clones derived from *Drosophila* spp. fruit flies [2]. Of these, 370 are derived from *D. melanogaster*, while just 16 cell lines are derived from 11 other species of *Drosophila*. Similarly, the Drosophila Genomics Resource Center website [3] lists 263 *Drosophila* spp. cell lines, sublines and clones – of these, all but 11 are derived from *D. melanogaster*. The Taxodros website lists over 1600 species in the genus *Drosophila* [4], so clearly there is a need for cell lines from a more diverse and representative panel of *Drosophila* spp.

In addition, we currently lack cell line representatives from any neotropical *Drosophila* spp., since all available lines are derived from either Old-World species native to Africa, Asia or Europe, or from Nearctic species. The Neotropical group of *Drosophila* flies is composed of 23 *willistoni* group species that evolved in isolation from Old World species over the last 40 million years in Central and South America. This isolation lasted until the sixteenth century when Old-World species such as *D. melanogaster* and *Drosophila simulans* co-invaded the American continent with human immigrants and settlers from Europe, Africa and Asia [5,6]. In addition, the neotropical *Drosophila* system has become famous as a genetic and ecological model for the mechanisms and dynamics of incipient speciation, i.e. as one of the rare systems of ongoing speciation in nature [7–11].

More recently, the *willistoni* group system has attracted broader attention because of their dominating role in serving as an ancestral host reservoir for the horizontal transfer of transposable elements (TEs) such as the *P* element DNA transposon [12,13] and the LTR transposon *Spoink* [14–16] into incoming Old World species such as *D. melanogaster* and *D. simulans*.

Furthermore, this neotropical system is also insightful for studying interactions between arthropods and reproductive bacterial symbionts such as *Wolbachia* [17–19] and *Spiroplasma* [20]. As with the two TEs mentioned above, *Wolbachia* and *Spiroplasma* were also both recently transmitted from neotropical hosts into invasive *melanogaster* group flies in nature [17,21]. This implies that neotropical *Drosophila* are the ancestral host reservoir for TEs and reproductive bacterial endosymbionts that have spread into invasive Old World *melanogaster* group flies during the colonization of the American continent by peoples of Old World origin since the sixteenth century. Therefore, this *Drosophila* system provides a unique opportunity to study long- and short-term dynamics of transposons and bacterial symbiotic infections within a 400-year time frame *in vivo*, as well as under artificial lab conditions such as by transinfection into heterologous cell lines *in vitro*.

*Wolbachia* is obligately intracellular, and has been reported to infect many different species of *Drosophila* [17,22,23], in which it has a range of biological effects [24–26]. However, we could find only two reports of *Drosophila* cell lines naturally and persistently infected with *Wolbachia*: the cell lines Dm2008Wb1 [27] and JW18 [28], both derived from *D. melanogaster*. As far as we know, only the JW18 cell line is publicly available from a cell culture collection (DGRC Stock Number: 356). Most laboratories work with *Drosophila* cell lines artificially transinfected with heterologous *Wolbachia* strains [29–31]. The lack of natural *in vitro* infection models, apart from the two *D. melanogaster* cell lines mentioned above, precludes comparisons between natural/homologous and transinfected/heterologous *Wolbachia* phenotypes in cell culture.

In the present study, we aimed to generate cell lines from the neotropical species *D. willistoni*, as well as to establish the *Wolbachia* strain *w*Wil in continuous *in vitro* culture in cells of its natural host. Here, we describe the generation of primary cell cultures from *D. willistoni* larvae, with subsequent establishment of three new continuous cell lines, and continuous cultivation of *w*Wil in homologous (naturally infected) and heterologous insect cell lines.

## Materials and Methods

### Flies

The *D. willistoni* flies were originally collected in February 2022 in La Gamba, Costa Rica along the Ocelot Trail (8.695369317701209, −83.2065482445985) of the Piedras Blancas National Park in Golfito, in agreement with the CONAGEBIO ID1185. Isofemale lines were established from inseminated generation 0 females and maintained in a laboratory colony at the Medical University of Vienna as described previously [19]. Adult flies at generation F6 were transported by air to the University of Liverpool in May 2022.

### Preparation of primary cell cultures

Eggs laid onto grape juice plates (prepared by adding 50 g agar, 600 ml grape juice and 42.5 ml of 10% Nipagin to 1 l of water, https://drosophoto.com/culturing-fruit-flies-2/#CFF_recipes) during the previous 16 h were rinsed with tap water in 70 μm Corning® cell strainers (Merck, Cat No. CLS431751), soaked in 0.1% benzalkonium chloride for 15 min and 70% ethanol for 1 min in a 30 mm plastic petri dish. The eggs were then rinsed with Shields and Sang M3 medium (Sigma, Cat No. S3652) supplemented with 20% foetal bovine serum, 2 mM L- glutamine, 100 units/ml penicillin and 100 µg/ml streptomycin (M3). Most of the eggs were gently crushed in 1 ml M3 with the flattened end of a glass rod to release the embryos, transferred to a 30 ml universal tube with an additional 2 ml M3 and centrifuged at 400 ×g for 5 min at room temperature. The supernatant medium was saved for *Wolbachia* isolation as described below, the cell pellet was resuspended in 0.4 ml M3 and the resultant tissue suspension was divided between two flat-sided culture tubes (Nunc, Cat No. 156758) with 1 ml of either M3 or Schneider’s *Drosophila* medium (Invitrogen, Cat No 21720024) supplemented with 20% foetal bovine serum, 2 mM L- glutamine, 100 units/ml penicillin and 100 µg/ml streptomycin (Schneider’s). The cultures were incubated at 22°C. Medium was changed weekly by removal and replacement of approximately half of the medium volume.

After 24–48 h incubation, larvae that hatched from uncrushed eggs in the primary cell cultures were transferred from the cultures into a petri dish and processed to generate larva-derived cultures. Larvae were either chopped in 0.1 ml M3 into 2–4 pieces using watchmaker’s forceps, and the resultant tissue suspension was transferred directly to the bottom of a new flat-sided tube, or were treated as follows. Larvae were chopped into 2–4 pieces in 0.1 ml HBSS, transferred with 1 ml HBSS to a universal tube and centrifuged for 5 min at 200 ×g. The tissue pellet was resuspended in 0.5 ml of 0.25% trypsin in PBS and incubated for 10–15 min at 28°C. An equal volume of complete medium (M3 or Schneider’s) was added, the suspension was centrifuged again, the pellet was resuspended in 0.2 ml complete medium and the tissue suspension was transferred to the bottom of a flat-sided tube. All larva-derived cultures were incubated at an angle of ~15° for the first 6 months to ensure that the tissues remained in close contact; thereafter the tubes were incubated horizontally.

### Cell line generation

Larva-derived cultures were incubated at 22°C for the first 4 weeks and thereafter at 26°C to encourage cell growth, with weekly removal and replacement of around half of the medium volume, and weekly examination by inverted microscope for signs of cell growth. When proliferating cells were observed, the medium volume was gradually increased over several months up to a final volume of 1.5 ml. Subculture was attempted when growing cells had covered at least 75% of the tube surface; cells were resuspended by pipetting and divided between the parent and one daughter culture. After 4–6 passages in flat-sided tubes, cells were transferred to T12.5 or T25 flasks (Falcon, Cat Nos 353018 and 353014), with 3 ml or 5 ml medium respectively. Cells were cryopreserved with 10% DMSO as described previously [32].

At intervals, Giemsa-stained cytocentrifuge smears were prepared from resuspended cultures as described previously [32], and examined for the presence of intracellular bacteria. To prepare *Wolbachia*-free sublines, cultures were treated with tetracycline hydrochloride (Sigma) at a final concentration of 5 µg/ml weekly for at least 8 weeks as described previously [33].

### Karyotyping

Subcultures prepared in T12.5 flasks from each cell line one day previously were treated with colcemid (Roche, Sigma-Aldrich, Cat No. 10295892001) at a final concentration of 1 µg/ml. After 17 h incubation, the cells were harvested, centrifuged at 400 ×g for 5 min at room temperature, the cell pellets were resuspended in 5 ml 0.5% trisodium citrate and the suspensions were incubated at 31°C for 40 min. The lysed cell suspensions were centrifuged as before; the resultant pellets of nuclei and cell debris were resuspended in ice-cold acetic alcohol (3 parts methanol, one part glacial acetic acid) and held on ice for 5 min. The centrifugation, resuspension and holding on ice were repeated once, the suspensions were again centrifuged and the pellets were resuspended in an equal volume of ice-cold acetic alcohol. Using a Pasteur pipette, the suspension was dropped from a height of 1 m onto ice-cold, wet glass slides. The slides were air-dried, stained with 3% Giemsa for 1 h, rinsed, air-dried and examined at × 500 oil immersion using a Leitz Orthoplan microscope. The chromosomes in 100 spreads from each cell line were counted.

### Confirmation of species origin and identification of Wolbachia

DNA was extracted from resuspended DWL/LULS68, DWL/LULS70 and DWL/LULS72 cells using a DNeasy Blood and Tissue kit (Qiagen, Cat No. 69504), following the manufacturer’s instructions for cultured cells. To confirm the species origin of the cells, a PCR targeting the invertebrate mitochondrial cytochrome c oxidase subunit I (COI), using primers LCO 1490 and HCO 2198, was carried out as described previously [34]. To confirm the identity of the *Wolbachia* in the cell lines, DNA extracted from cultured cells was subjected to conventional PCRs amplifying fragments of the pan-bacterial 16S rRNA gene [35] and the *Wolbachia wsp* gene [36] following the respective published protocols. The PCR products were visualized by agarose gel electrophoresis. Positive PCR products were purified using a PureLink Quick Gel Extraction and PCR Purification Combo kit (Thermo Fisher, Cat No. K220001) following the manufacturer’s instructions, and Sanger-sequenced in both directions (Eurofins Genomics, Ebersberg, Germany). The chromatograms were analysed using 4peaks (https://nucleobytes.com/4peaks/). Alignments were performed using AliView to obtain consensus sequences [37]. The COI sequences from each cell line were deposited in Genbank under accession numbers PX981764 (DWL/LULS68), PX981765 (DWL/LULS70) and PX981766 (DWL/LULS72).

### Isolation of Wolbachia strain wWil and growth in heterologous insect cell lines

To isolate *Wolbachia* directly from *D. willistoni* embryos, supernatant medium saved during the initial preparation of eggs (see above) was centrifuged at 2,000 ×g for 5 min at room temperature to ensure any intact cells were pelleted and 0.5 ml aliquots of the resultant cell-free supernate were added to cultures of each of two sand fly larva-derived cell lines (PPL/LULS49 and LLL/LULS52) and two embryo-derived tick cell lines (BME/CTVM23 and IDE8) (Table 1). The inoculated cultures were incubated at 28°C. To test infectivity of *w*Wil for additional heterologous cell lines, pooled supernatant medium from DWL/LULS68 cell cultures at passage 1–3 was centrifuged for 5 min at 400 ×g, passed through a 0.45 µm filter, and 0.5 ml was inoculated into each of three sand fly, four midge, two triatomine bug and one tsetse fly cell lines (Table 1). All inoculated cultures were incubated at 26°C apart from the tsetse fly cells that were incubated at 28°C. Medium was changed weekly, Giemsa-stained cytocentrifuge smears were prepared and examined for *Wolbachia* at intervals, and all cultures that did not reveal intracellular bacteria were screened by a qPCR targeting the *Wolbachia* 16S rRNA gene as described previously [45].

**Table 1.** Insect and tick cell lines tested for ability to support isolation, infection and growth of *Wolbachia w*Wil derived from *Drosophila willistoni* embryos and the larva-derived cell line DWL/LULS68.

Cell line	Species	Reference	*w*Wil growth
**Inoculated with supernate from *D. willistoni* embryos**			
LLL/LULS52	*Lutzomyia longipalpis* (Sobral)	Bell-Sakyi et al. [33]	++
PPL/LULS49*	*Phlebotomus papatasi*	Bell-Sakyi et al. [33]	–
BME/CTVM23	*Rhipicephalus microplus*	Alberdi et al. [38]	–
IDE8	*Ixodes scapularis*	Munderloh et al. [39]	–
**Inoculated with supernate from *D. willistoni* cell line DWL/LULS68**			
LLL/LULS52	*L. longipalpis* (Sobral)	Bell-Sakyi et al. [33]	++
LLE/LULS40	*L. longipalpis* (Campo Grande)	Bell-Sakyi et al. [40]	+++
LLE/LULS45	*L. longipalpis* (Jacobina)	Bell-Sakyi et al. [33]	–
CNE/LULS44	*Culicoides nubeculosus*	Bell-Sakyi et al. [32]	–
CNE/LULS47	*C. nubeculosus*	Bell-Sakyi et al. [32]	–
KC cells	*Culicoides sonorensis*	Wechsler et al. [41]	+++
CSL/LULS64	*C. sonorensis*	Hartley et al. [42]	++
RPE/LULS53	*Rhodnius prolixus*	Penrice-Randal et al. [43]	–
TIE/LULS54	*Triatoma infestans*	Penrice-Randal et al. [43]	–
GMA/LULS61	*Glossina morsitans morsitans*	Bell-Sakyi et al. [44]	++

*The PPL/LULS49 subline previously cured of infection with *Wolbachia w*Pap by tetracycline treatment [33] was used.

### Fluorescence in situ hybridization (FISH) to detect intracellular Wolbachia

For detection of *Wolbachia* in cell cultures by FISH, ~5 × 10^5^ cells were harvested and centrifuged at 500 × g for 5 min to pellet the cells. Most of the medium was discarded with 0.2 ml left in the tube to prevent the pellet from drying. The cells were gently resuspended and 2 mL of fixative 1 (3.7% paraformaldehyde in complete culture medium used) was added and mixed gently by inversion. The cells were incubated at room temperature for 10 min with shaking at ~ 20 oscillations per min, and then centrifuged as above. The supernatant was removed and 2 mL of fixative 2 (3.7% paraformaldehyde in 0.1 M PBS) was added. The pellet was resuspended, and cells were incubated at room temperature for 10 min shaking as above, with subsequent centrifugation at 500 × g for 5 min. The supernatant was removed, and the pellet was resuspended in 2 ml of 0.1 M PBS for rinsing. The rinsing with 0.1 M PBS was repeated two more times. At the last rinsing step, the cell suspension was distributed into 1.5 ml tubes and centrifuged at 500 × g for 5 min. The supernate was removed, and 80% ethanol added. The cells were resuspended in ethanol and held at 4°C overnight before being shipped by courier to the Medical University of Vienna; the journey took six days, during which the samples were subjected to ambient temperature. On arrival in Vienna, the samples were stored at 4°C for up to two weeks before being processed for FISH.

For FISH, cells were rehydrated in a series of buffers with gradually decreasing ethanol in 0.1 M PBS (ethanol:PBS 50:50, 30:70, 10:90, 0:100). Then 20 µl drops of cell suspension were transferred to microscope slides and spread out using rectangular coverslips in a manner similar to that used to prepare thin blood smears. After 20 min of drying at room temperature, cells were first hydrated with a 50 µl drop of washing buffer (10% formamide in 4x saline sodium citrate prepared from SSC buffer 20x concentrate, Sigma Cat. No. S6639) and then incubated at 37°C for 3 h in a drop of hybridization buffer (10% formamide and 10% dextran sulphate in 4x SSC), containing 0.5 nmol of CAL Fluor Red 590-labelled Stellaris *Wolbachia 16-23S* rRNA probe (custom-made by Biosearch Technologies [46]). Samples were rinsed with drops of the washing buffer 3 times, incubated in the same buffer for 30 min at 37°C, covered with mounting medium containing DAPI (ROTI®Mount FluorCare, Carl Roth, Cat. No. HP20.1) and closed with a coverslip. The cells were viewed on an Olympus FluoView FV3000 confocal microscope.

### Drosophila cell line phylogenetic tree construction

For each species, we chose the best assemblies available in NCBI (accessed July 2025), based on the assembly level status, and the scaffold N50. Genome assemblies (GCA_018903445.1, GCA_016746365.2, GCA_016746235.2, GCA_003286155.2, GCA_004382185.1, GCA_004382195.2, GCA_003401975.1, GCA_008042615.1, GCA_963583985.1, GCA_009870125.2, GCA_000004125.1, GCA_027580165.1, GCA_963583835.1, and GCA_004354385.2) were downloaded from NCBI and analysed with BUSCO (v5.0.0 [47]) in order to identify single orthologs. We found a total of 2,648 genes identified as single orthologs in all species. For each gene, the corresponding protein sequences were aligned with mafft v7.505 (-retree1 [48]). Alignments were cleaned using TrimAl (-automated1 [49]). Three hundred alignments, randomly selected among the 2,648 alignments, were concatenated into a supermatrix, representing 201,274 residues (44,920 parsimony-informative residues). The tree was constructed by maximum likelihood using IQ-TREE3 [50] with the Q.INSECT+F+I+R3 evolution model as selected (based on the BIC scores) by ModelFinder [51]. Robustness was assessed with Ultrafast Bootstrap [52]. The topology of the resulting tree was congruent with the phylogeny in Suvorov et al. [53] and all nodes had 100% bootstrap support. Calibration was done by ultrametrization (R package ape [54]), with the root (identified by a black circle) constrained within between 43 and 51 MYR, based on data available in Suvorov et al. [53].

## Results

### D. willistoni primary embryo- and larva-derived cell cultures

A total of five embryo-derived primary cultures were set up in flat-sided culture tubes using eggs laid on two successive days. Two tubes were set up in each of M3 and Schneider’s media, and a single tube was set up in H-Lac medium. Initially, some clumps of growing cells appeared in these cultures, but sustained cell multiplication did not ensue. Many uncrushed eggs hatched into larvae that were removed from the cultures after 1–14 days’ incubation and processed for larva-derived primary cultures as described below. All embryo-derived cultures were discarded by day 233 when no viable cells remained.

**Figure 1.**
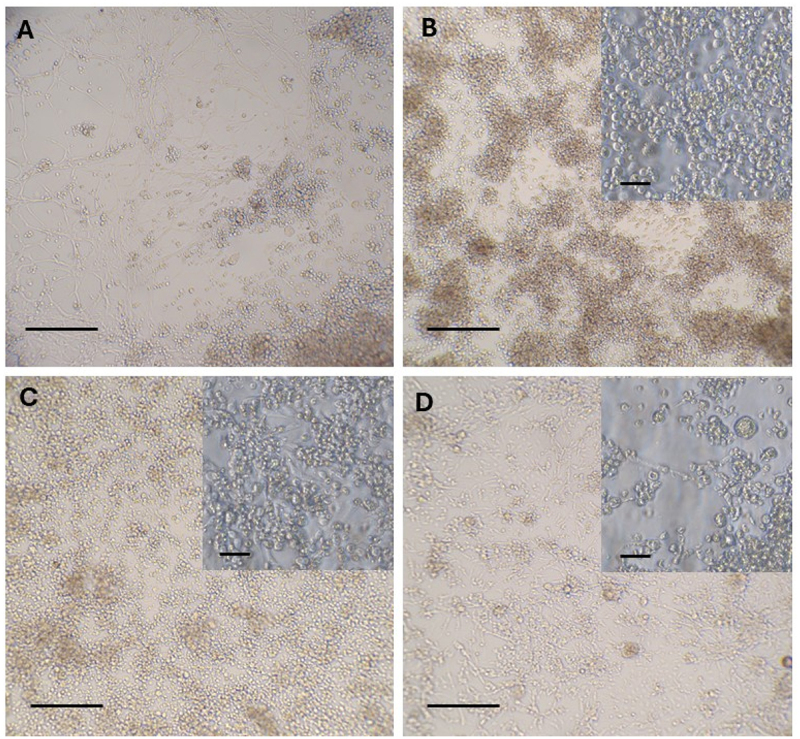
Light microscopic morphology of *Drosophila willistoni* primary cell cultures and cell lines. (A) Primary cell culture 12 months post initiation. (B) DWL/LULS68 cells at passage 13, 20 months post initiation. (C) DWL/LULS70 cells (tetracycline-treated subline) at passage 15, 29 months post initiation. (D) DWL/LULS72 cells at passage 7, 29 months post initiation. Main pictures: live, inverted microscope; scale bars = 200 µm. Insets: live, phase contrast inverted microscope; scale bars = 20 µm.

Three larva-derived primary cultures were set up in flat-sided tubes with between 13 and 25, 1–2-day old larvae removed from the embryo-derived cultures. Two tubes were set up in M3 medium, while the third was initiated in Schneider’s medium and transferred to M3 after 228 days. Small patches of growing round or spindle-shaped cells were first observed in the cultures from day 6 onwards, but sustained cell growth did not begin until the larval tissues had been in culture for at least 200 days (Figure 1(A)). Despite this extended lag phase, initiation of primary cultures from larvae was thus found to be a successful approach.

### Establishment of the DWL/LULS68, DWL/LULS70 and DWL/LULS72 cell lines

Continuous cell lines, designated DWL/LULS68, DWL/LULS70 and DWL/LULS72 (Table 2), were successfully generated from each of the three larva-derived primary cultures. First subcultures were carried out between 12 and 16 months after initiation, with successful cryopreservation confirmed at passage levels between 2 and 11, 14–33 months after initiation (Table 2). Once established, the cells could be maintained in T12.5 or T25 flasks and passaged routinely once every two weeks, with split ratios between 1:1 and 1:4 as required. The cell lines are all phenotypically heterogeneous, comprising predominantly round, floating or adherent cells, and smaller numbers of adherent spindle-shaped cells (Table 2, Figure 1(B, C, D)).

**Table 2.** Characteristics of the three larva-derived *Drosophila willistoni* cell lines DWL/LULS68, DWL/LULS70 and DWL/LULS72.

Cell line	DWL/LULS68	DWL/LULS70	DWL/LULS72
Starting material	1-day-old larvae (*n* = 13)	1–2-day old larvae (*n* = 17)	1–2-day-old larvae (*n* = 25)
Culture medium	M3*	M3	Schneiders** up to day 199; M3 from day 200
Age at first passage	12 months	14 months	16 months
Age/passage level (p) at first cryopreservation	14 months/p2	22 months/p7	33 months/p11
Passage interval following establishment	2 weeks (1:1 – 1:4)	2 weeks (1:1 – 1:4)	2 weeks (1:1 – 1:4)
Cell phenotype	Predominantly round, partly attached, partly floating, with a tendency to clump	Mix of predominantly round, floating and adherent cells, few adherent spindle-shaped cells	Mix of mostly adherent, round, fibroblast-like and spindle-shaped cells
Cell dimensions	Mean diameter 8.21 µm (range 4.02–24.89 µm)	Mean diameter 7.58 µm (range 4.06–17.41 µm)	Mean length 13.56 µm (range 4.19–69.98 µm)

*Shields and Sang M3 medium supplemented with 20% foetal bovine serum (FBS), 2 mM L-glutamine, 100 units/ml penicillin and 100 µg/ml streptomycin.

** Schneider’s *Drosophila* medium supplemented with 20% FBS, 2 mM L-glutamine, 100 units/ml penicillin and 100 µg/ml streptomycin.

**Figure 2.**
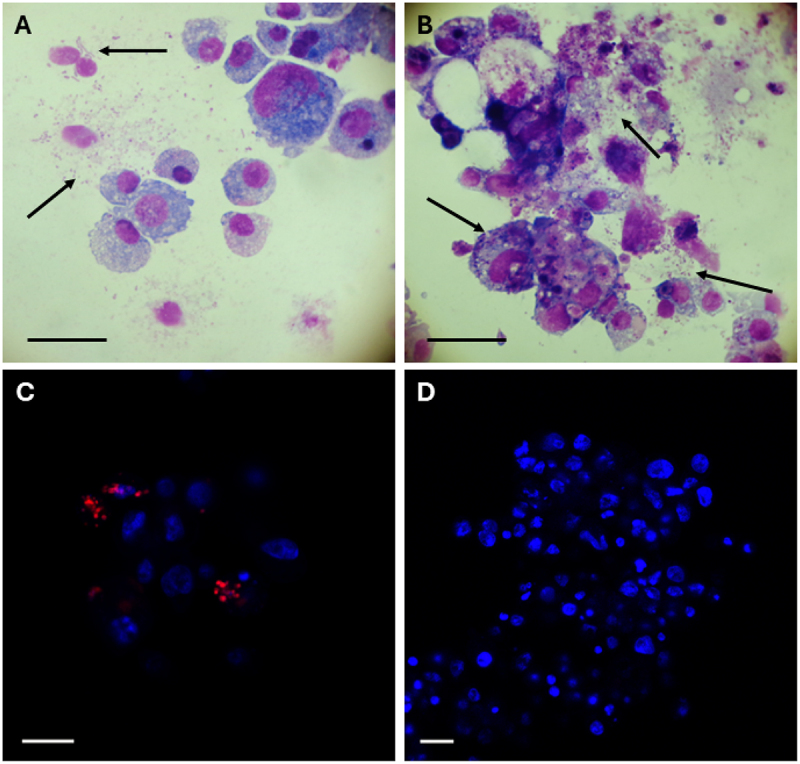
*Wolbachia w*Wil growing in *Drosophila willistoni* cell lines. (A) DWL/LULS68 at passage 3 and (B) DWL/LULS72 at passage 12; Giemsa-stained cytocentrifuge smears; arrows indicate *Wolbachia*; scale bars = 20 µm. FISH staining of (C) *w*Wil-infected DWL/LULS68 cells at passage 13 and (D) DWL/LULS68 cells cured of *w*Wil infection by prolonged tetracycline treatment at passage 13; red staining = CAL fluor red 590-labelled stellaris *Wolbachia 16-23S* rRNA probe, blue = DAPI; scale bars = 10 µm.

Giemsa-stained cytocentrifuge smears prepared from early subcultures confirmed the presence of intracellular bacteria in all three cell lines, with 16–70% of cells visibly infected (Figure 2(A,B)). Subsequent PCR-screening of DNA extracted from early subcultures of DWL/LULS68, DWL/LULS70 and DWL/LULS72, at 16, 16 and 26 months post-initiation respectively, confirmed identity of the bacteria as *Wolbachia* strain *w*Wil (100% similarity with *Wolbachia* endosymbiont of *D. willistoni* isolate SC20230912, query cover 100%, Genbank accession no. CP157591.1). FISH staining specific for *Wolbachia* carried out on later passages of DWL/LULS68 (Figure 2(C)) and DWL/LULS70 cells revealed infection rates of around 50%, with variable infection levels per cell. Sublines of DWL/LULS68 and DWL/LULS70 without *w*Wil were successfully generated by tetracycline treatment for 10 weeks; absence of *Wolbachia* was confirmed by FISH (Figure 2(D)) and *Wolbachia* qPCR carried out on DNA extracted 1–11 weeks following cessation of treatment. A similar tetracycline treatment regime reduced but failed to completely remove the *w*Wil infection from DWL/LULS72 cells; continued treatment for a further 11 weeks successfully removed the remaining *Wolbachia* as confirmed by failure to amplify any product by conventional or qPCR. At the time of writing, this subline has reached passage 35.

**Figure 3.**
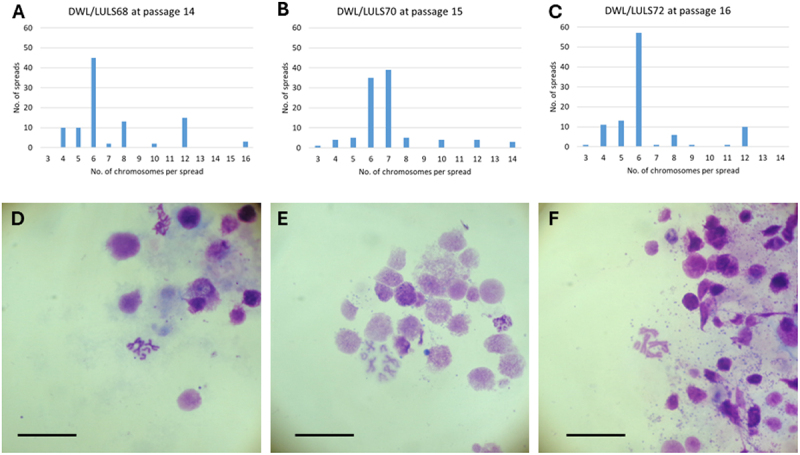
Karyotype analysis of *Drosophila willistoni* cell lines DWL/LULS68 (A) and (D), DWL/LULS70 (B) and (E) and DWL/LULS72 (C) and (F). (A-C) Distribution of chromosome numbers in 100 metaphase chromosome spreads. (D-F) examples of metaphase chromosome spreads with diploid numbers of five, seven and six chromosomes respectively; Giemsa stain, scale bars = 10 μm.

Karyotype analysis was carried out on the original *Wolbachia*-infected cell lines at passages 14, 15 and 16 of DWL/LULS68, DWL/LULS70 and DWL/LULS72, respectively. Chromosome spreads with the expected diploid complement of six – two large metacentric autosomes, two small acrocentric autosomes and two submetacentric sex chromosomes [55] – were present in all three cell lines, and formed the majority of spreads in DWL/LULS68 (45%, Figure 3(A)) and DWL/LULS72 (57%, Figure 3(C,F)). In DWL/LULS70, however, spreads with seven chromosomes slightly outnumbered spreads with six chromosomes (39% versus 35%, Figure 3(B)); in those spreads in which chromosome size and morphology could be clearly seen, the seventh chromosome was always an extra small acrocentric chromosome (Figure 3(E)). Spreads with fewer than the expected diploid number either lacked one of the large chromosomes (Figure 3(D)) or both of the small chromosomes (not shown). Where chromosome morphology could be distinguished, some spreads in DWL/LULS68 appeared to be male, with presence of a darker-staining, more condensed male chromosome [55], while other spreads in this line, and both the other lines, appeared female with all chromosomes showing similar staining intensity.

Species origin of all three cell lines was confirmed by insect COI PCR carried out on DNA extracted from both *w*Wil-infected and tetracycline-treated cells. The resulting 658 bp sequences, all with 100% query cover, showed 100% identity with *D. willistoni* strain FG168 mitochondrion from French Guiana (Genbank accession number OP208747.1) for DWL/LULS68, 99.85% identity with *D. willistoni* strain APA8 mitochondrion from Mexico (Genbank accession number NC_071240.1) for DWL/LULS70, and 99.85% identity with *D. willistoni* haplotype 2 mitochondrion from Brazil (Genbank accession number JN705921.1) for DWL/LULS72. The three COI sequences were not identical, with one and two nucleotide differences, respectively, between DWL/LULS70 and DWL/LULS72 and between DWL/LULS68 and DWL/LULS70.

The three new cell lines, while all derived from *D. willistoni* larvae originating from the same geographical location and all available as uninfected and *Wolbachia*-infected sublines, each have slightly different phenotypes and may thus have differing applications for specific research areas.

### Growth of wWil in heterologous cell lines

**Figure 4.**
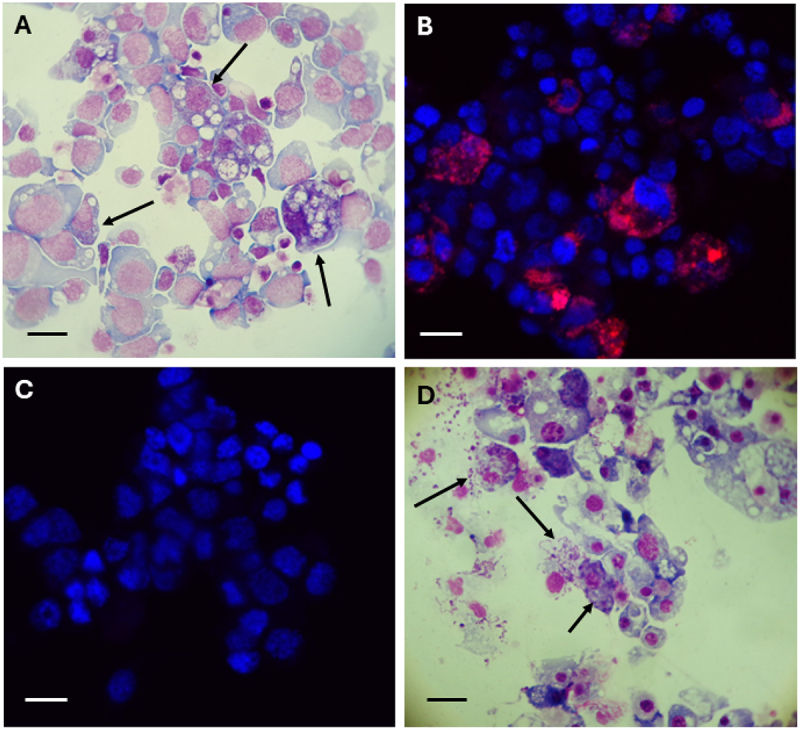
*Wolbachia w*Wil growing in heterologous insect cell lines. *Lutzomyia longipalpis* cell line LLE/LULS52 infected with *w*Wil from cell-free supernate generated during initiation of *Drosophila willistoni* embryo-derived primary cell cultures (A) Giemsa-stained cytocentrifuge smear; arrows indicate *Wolbachia*; (B) FISH staining of wWil-infected LLL/LULS52 cells carried out 11 months after initial inoculation, red staining = CAL fluor red 590‑labelled stellaris *Wolbachia 16-23S* rRNA probe, blue = DAPI; (C) FISH staining of uninfected control LLL/LULS52 cells; (D) *Culicoides sonorensis* cell line KC transinfected with *w*Wil from DWL/LULS68. Arrows indicate *Wolbachia*; scale bars = 10 µm.

An initial attempt was made to isolate *Wolbachia* from cell-free supernate collected during the generation of embryo-derived primary cell cultures. Supernate from two separate culture attempts was inoculated into two insect and two tick cell lines: *L. longipalpis* LLL/LULS52, *P. papatasi* PPL/LULS49, *R. microplus* BME/CTVM23 and *I. scapularis* IDE8, all previously shown to support growth of other *Wolbachia* strains [33,56]. The inoculum caused severe cytopathic effect in both tick cell lines within 48 h, and these cultures were discontinued. The recipient sand fly cultures were maintained for at least 15 weeks p.i., with periodic examination of Giemsa-stained smears for presence of *Wolbachia*-infected cells. At 13 weeks p.i., *Wolbachia*-infected cells were first seen in both recipient LLL/LULS52 cultures, with a gradual increase in the proportion of infected cells over the subsequent four weeks. Initially, bacteria were seen only in large, vacuolated cells, but after several month in LLL/LULS52 culture, bacteria were also seen in smaller cells with homogeneous cytoplasm (Figure 4(A)). The infection in both LLL/LULS52 cultures was confirmed as *w*Wil by sequencing of *wsp* PCR products amplified from DNA extracted at 15 weeks. The FISH staining carried out on *w*Wil-infected LLL/LULS52 cells at passage 1, 11 months after the initial infection of this cell line, revealed specific staining of bacteria in infected cells (Figure 4(B)), while no specific staining was seen in uninfected control LLL/LULS52 cells (Figure 4(C)). No bacteria were seen in either of the recipient PPL/LULS49 cultures throughout the observation period, and both were negative for *Wolbachia* when tested by *Wolbachia* 16S rRNA qPCR at 15 weeks.

Susceptibility of the LLL/LULS52 cell line to *w*Wil was confirmed by inoculation of cell-free, unfiltered supernate from the primary *D. willistoni* culture that subsequently yielded the DWL/LULS68 cell line, 3.5 months after initiation. Again, intracellular bacteria were not seen until 13 weeks p.i.

To investigate whether *w*Wil could infect and grow in additional insect-derived cell lines, cell-free filtered supernate from DWL/LULS68 cultures at passage 1–3, 13 months after initiation, was added to a panel of previously untested cell lines and LLL/LULS52 as a control. The panel comprised two cell lines each derived from the biting midges *Culicoides nubeculosus* and *Culicoides sonorensis*, one each from the triatomine bugs *Rhodnius prolixus* and *Triatoma infestans* and from the tsetse fly *Glossina morsitans morsitans*, and two additional cell lines derived from different laboratory strains of *L. longipalpis* (Table 1). The cultures were sampled by preparation of cytocentrifuge smears at 2–3 week intervals, but infection was not detected until the twelfth or thirteenth week, at which point heavy infections were seen in LLE/LULS40 and KC cells (Figure 4(D)) and light infections in LLL/LULS52, CSL/LULS64 and GMA/LULS61 cells. Some cytopathic effect (CPE) was seen in the infected GMA/LULS61 cells, while the other four cell lines supported infection with *w*Wil through up to four subcultures in the absence of CPE, before being cryopreserved nine months after initial inoculation. Intracellular bacteria were not seen in LLE/LULS45, either of the *C. nubeculosus* cell lines, or either of the triatomine bug cell lines at any time, and all five cell lines were negative by *Wolbachia* qPCR at 6 months. Thus, *w*Wil was able to infect and grow in some, but not all, of the tested cell lines derived from heterologous arthropod taxa, making it a promising candidate for elucidating *Wolbachia*-host cell interactions.

### Phylogeny of Drosophila spp. from which cell lines are available

**Figure 5.**
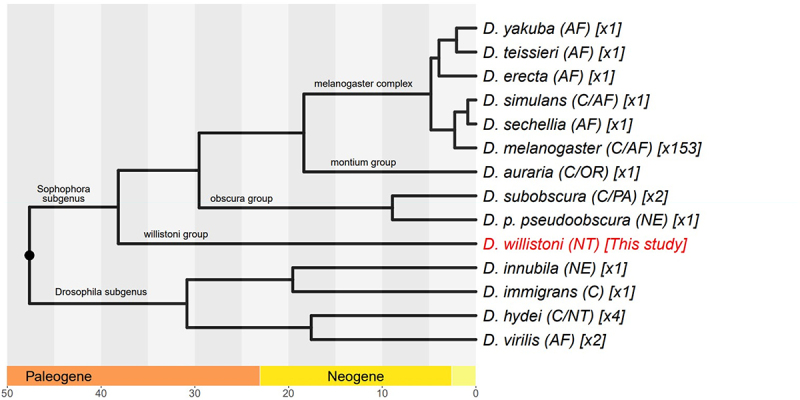
Phylogenetic relationships of *Drosophila* species for which cell lines are available. The tree was constructed from a supermatrix made of 300 protein sequences from core genes (BUSCO) using IQ-TREE3 as described in Materials and Methods. All nodes had 100% bootstrap support. The geographic distributions/origins of the species are shown in parentheses (AF: Afrotropical, AU: Australasian, NE: Nearctic, NT: Neotropical, PA: Palaeoarctic, C: Cosmopolitan). The number of parent cell lines derived from each species is indicated in square brackets. Time is indicated in millions of years on the X-axis.

Phylogenetic analysis of *D. willistoni* and the other 13 *Drosophila* spp. from which cell lines have been generated placed the *willistoni* group within the subgenus Sophophora, and confirmed its separation from other groups within this subgenus around 40 million years ago (Figure 5). Consequently, the three *D. willistoni* cell lines, generated from recently collected flies at F6, represent a novel system for *in vitro* study of a non-*melanogaster* Neotropical *Drosophila* species and its native *Wolbachia* endosymbiont that have both adapted to tropical environments in the New World.

## Discussion

Amongst the nearly 370 published *Drosophila* spp. cell lines, sublines and clones, over 95% are derived from *D. melanogaster*, and just under 90% are embryo-derived. Our three new larva-derived *D. willistoni* cell lines therefore contribute significantly to the diversity of *Drosophila* cell line resources, both by adding to the 12 non-*melanogaster* species (17 cell lines) currently available, and by increasing the number of cell lines derived from instars other than embryos. Moreover, we have more than doubled the numbers of available *Drosophila* cell lines naturally infected with *Wolbachia*, and added a new natural host species, *D. willistoni*.

The method used to generate the *D. willistoni* primary cultures differed from the generally recommended approaches for *Drosophila* embryos [57,58]. In particular, surface sterilization was achieved using 0.1% benzalkonium chloride and 70% ethanol rather than bleach, and no attempt was made to dechorionate the eggs prior to crushing them. It is unclear whether either of these deviations was responsible for the failure to obtain growth of embryo-derived cells, or if the failure was due to some intrinsic characteristic of *D. willistoni*. The method used to generate the larva-derived primary cultures that eventually gave rise to the three cell lines DWL/LULS68, DWL/LULS70 and DWL/LULS72 also differed from that used by Cullen and Milner [59] to generate *D. melanogaster* larva-derived cultures. Those authors dissected out imaginal discs from axenically raised third instar larvae, whereas in the present study, 1–2-day-old larvae hatched from uncrushed eggs in the embryo-derived primary cultures were simply chopped into several pieces and maintained in culture medium. Again, it is unclear whether the different approach was responsible for the delay in the start of *D. willistoni* cell growth; Cullen and Milner [59] reported cell growth within the first few days and carried out their first subcultures between 10 and 50 days post initiation, whereas the first *D. willistoni* subcultures were not carried out until 12–16 months post initiation. We have previously used the simple approach of chopping up larvae, hatched from uncrushed eggs in primary cultures, to generate cell lines from the sand flies *L. longipalpis*, *P. papatasi* and *Phlebotomus argentipes*, the mosquitoes *Culex pipiens molestus* and *Anopheles stephensi*, and the biting midge *C. sonorensis* [33,42]. In all cases, larva-derived cells did not start to grow immediately, and took between 6 and 34 months to reach a stage when they could be subcultured.

All three cell lines were confirmed to be of *D. willistoni* origin by molecular analysis, and karyotyping revealed that the majority of cells in two of the lines had either the expected diploid number of six chromosomes [55], or were tetraploid with 12 chromosomes. Interestingly, in DWL/LULS70, nearly two-fifths of the cells had seven chromosomes. Due to the constraints of the karyotyping method used, it was not possible to determine chromosome morphology in many of the spreads, but in those where morphology was distinguishable, the seventh chromosome consistently appeared to be an extra copy of the small acrocentric chromosome. Polyploidy and karyotype evolution over time during *in vitro* culture have been reported previously for cell lines derived from *D. melanogaster* [60,61]; in those cases, translocations and/or tetraploidy followed by rearrangements or loss of partial or entire chromosomes were proposed as mechanisms to explain the observed karyotypes. It is not clear whether the extra small autosome observed in some DWL/LULS70 cells arose as a result of duplication of a single chromosome *in vitro*, or whether one or more of the *D. willistoni* larvae from which the cell line was derived may have carried an extra chromosome. The latter scenario, although unlikely, is not unknown amongst dipteran arthropods; we could not find examples in the literature of extra autosomes amongst field collected *Drosophila* spp., but variable autosome numbers have been reported for *Glossina* spp. tsetse flies [62,63]].

As mentioned above, we could only find two published *Drosophila* cell lines naturally and persistently infected with *Wolbachia*, both derived from *D. melanogaster* [27,28]. It has been speculated that natural *Wolbachia* infections could assist in the successful generation of *Drosophila* primary cultures and cell lines [57]. Certainly, in the present study, we did not find any deleterious effect caused by *w*Wil infection in the *D. willistoni* cells while, at the time of writing, infected and tetracycline-treated cells of all three cell lines grow at similar rates and exhibit similar morphological phenotypes. This observation implies either that *w*Wil does not provide *D. willistoni* cells with essential nutrients, at least *in vitro*, or that all nutritional needs of the cells are adequately provided by the M3 medium and FBS. Moreover, *in vivo*, *w*Wil has evolved quite tight and well-defined tissue tropisms in its native host by targeting mainly embryonic primordial germ line cells, i.e. the future gonad [17], and only a small subset of somatic neuroblasts [64,65]. In contrast, in the *w*Mel-*D. melanogaster* endosymbiosis and most other arthropod infection associations, the native *Wolbachia* are more globally distributed throughout the host tissues and are hence systemic with no defined tropism. In conjunction with the recently published *w*Wil genome assembly generated from bacteria transinfected from *D. willistoni* embryos into the *D. melanogaster* cell line JW18 [66], availability of the three *D. willistoni* cell lines naturally infected with *w*Wil, as well as the uninfected sublines, will facilitate detailed studies of the interaction between symbiont and host.

Transinfection of *Wolbachia* into heterologous *Drosophila* cell lines (not derived from the natural invertebrate host) has previously been achieved using the shell vial technique, which employs medium-speed centrifugation to force the bacteria onto the cells [66,67]. Working with a range of *Wolbachia* strains and arthropod cell lines, we have generally found it sufficient to add a suspension of cell-free bacteria to the medium overlaying the recipient cells [33,44,56]; if the cells are susceptible, they will take up the bacteria. In the present study we successfully transferred *w*Wil from *D. willistoni* embryos of cultured larva-derived cells to cell lines derived from the New World sand fly *L. longipalpis*, the New World biting midge *C. sonorensis* and the African tsetse fly *G. m. morsitans*, but failed to infect cell lines derived from the Old World sand fly *P. papatasi*, the Old World biting midge *C. nubeculosus*, the New World triatomine bugs *R. prolixus* and *T. infestans* and two tick species. We cannot rule out the possibility that the latter cell lines might become infected if subjected to the shell vial approach; this could be tested in future experiments. All the dipteran cell lines tested apart from KC and CSL/LULS64, and both tick cell lines, have previously been shown to support either natural [33] or transinfected [33,44,56] *Wolbachia* infections; other cell lines derived from *C. sonorensis* were shown to support *Wolbachia* infection [68]. While *Wolbachia* 16S rRNA sequences have been detected at low abundance in *T. infestans* [69], there are no published reports of propagation of any *Wolbachia* strains in triatomine cell lines. It will be of interest to determine whether or not *w*Wil can infect and proliferate in cell lines derived from other *Drosophila* spp., in particular *D. melanogaster* lines used as well-established models for studying interactions with *Wolbachia*, such as S2 and JW18 [67,70,71].

To conclude, these new cell lines will allow study, in their native genomic background, of the structural and functional, short- and long-term dynamics of *Wolbachia* symbionts and other components of the bacteriome of Neotropical *Drosophila* spp., such as *Spiroplasma*, as well as their native transposable elements known as the mobilome. Moreover, coinfections with different microbial or transposon strains and variants plus their reciprocal transfer between native (homologous) and foreign (heterologous) cell line backgrounds will help to improve our understanding of the complex interplay of cooperation and competition between different selfish genetic elements and their hosts under *in vitro* conditions. The three *D. willistoni* cell lines DWL/LULS68, DWL/LULS70 and DWL/LULS72, derived from larvae by a relatively simple approach, and available as sublines with and without natural infections with *Wolbachia* strain *w*Wil, can be sourced by the international *Drosophila* research community from the Tick Cell Biobank at the University of Liverpool (https://www.liverpool.ac.uk/research/facilities/tick-cell-biobank/), subject to an appropriate Material Transfer Agreement.

## Data Availability

The data that support the findings of this study are openly available in Genbank, accession numbers PX981764-PX981766: https://www.ncbi.nlm.nih.gov/search/all/?term=PX981764 https://www.ncbi.nlm.nih.gov/search/all/?term=PX981765 and https://www.ncbi.nlm.nih.gov/search/all/?term=PX981766
